# Trends in Disability-Free Life Expectancy in Japan, 1995–2004

**DOI:** 10.2188/jea.JE20090190

**Published:** 2010-07-05

**Authors:** Shuji Hashimoto, Miyuki Kawado, Rumi Seko, Yoshitaka Murakami, Masayuki Hayashi, Masahiro Kato, Tatsuya Noda, Toshiyuki Ojima, Masato Nagai, Ichiro Tsuji

**Affiliations:** 1 Department of Hygiene, Fujita Health University School of Medicine, Toyoake, Japan; 2 Faculty of Nursing, Fujita Health University School of Health Sciences, Toyoake, Japan; 3 Department of Medical Statistics, Shiga University of Medical Science, Otsu, Japan; 4 Department of Information Science, Fukushima Medical University School of Nursing, Fukushima, Japan; 5 Seto Public Health Center, Aichi Prefecture, Seto, Japan; 6 Department of Community Health and Preventive Medicine, Hamamatsu University School of Medicine, Hamamatsu, Japan; 7 Division of Epidemiology, Department of Public Health and Forensic Medicine, Tohoku University Graduate School of Medicine, Sendai, Japan

**Keywords:** disability-free life expectancy, healthy life expectancy, health statistics

## Abstract

**Background:**

In Japan, life expectancy at birth is currently the highest in the world. However, recent trends in disability-free life expectancy in Japan have not been examined.

**Methods:**

We used data from Japanese national surveys for the period 1995–2004. These surveys included information on activity status measured by common self-reported instruments. The numbers of expected years with and without activity limitation were estimated by using the Sullivan method.

**Results:**

The numbers of expected years of life without activity limitation, at birth, in 1995 and 2004 were 68.5 and 69.7, respectively, in males and 72.1 and 73.0 in females. As a proportion of total life expectancy, at birth, these values represent a decrease from 89.7% to 88.6% in males and from 87.1% to 85.3% in females. The proportion of expected years with a limitation of some activities except activities of daily living (ADL) increased in males and females. The proportion of those with an ADL limitation increased in females, but not in males.

**Conclusions:**

The trends in expected years with and without activity limitation suggest that the duration of life with a light or moderate disability increased in Japanese males and females during the period 1995–2004.

## INTRODUCTION

Although life expectancy provides an estimate of average expected lifespan, health expectancy partitions total life expectancy into years free from health-related problems and years lived in ill-health.^1^^,^^2^ Two measures of health expectancy are widely used: disability-free life expectancy is defined as expected years of life without disability or activity limitation, and healthy life expectancy is based on self-rated health.^1^^,^^3^^,^^4^

The trends in disability-free life expectancy observed in several countries indicate that increases in total expected years of life are not necessarily accompanied by increases in the expected years of, or proportion of, active life.^5^^–^^8^ In Japan, life expectancy at birth is now the longest in the world, and continues to increase.^9^^–^^11^ Healthy life expectancy has been reported,^12^ but there have been no reports of recent trends in disability-free life expectancy, as determined using national survey data.^1^^,^^13^

In the present study, the expected years with and without activity limitation for the period 1995–2004 were estimated in Japanese by using data from national surveys that included information on activity status, as measured by common instruments.

## METHODS

### Data

We used data from life tables and the population in Japan during the period 1995–2004.^10^^,^^11^ Data on activity status for persons living at home were obtained from the Comprehensive Survey of Living Conditions of the People on Health and Welfare of 1995, 1998, 2001, and 2004, which was a self-administered questionnaire survey distributed to more than 750 000 persons in households randomly selected nationwide.^14^

The data for patients admitted to hospitals and clinics were from the Patient Survey of 1996, 1999, 2002, and 2005, which was distributed to more than 3 000 000 patients who visited hospitals and clinics randomly selected throughout Japan.^15^ Before 1999, data for elder Japanese admitted to facilities for the health of older adults who need nursing care were obtained from the Survey on Health Service Facilities for the Aged, and data on elders admitted to welfare facilities for older adults who need nursing care were obtained from the Survey of Social Welfare Institutions. After 2000, both these datasets were collected from the Survey of Institutions and Establishments for Long-term Care.^16^

### Activity limitation

The activity status for persons living at home was evaluated using responses to the questions: “Is your daily life now affected by health problems?” and “How is it affected?”.^14^ The second question was for persons replying “Yes” to the first question. The responses to the second question were “activities of daily living (ADL) (rising, dressing/undressing, eating, bathing, etc),” “going out,” “work, housework or schoolwork,” “physical exercise (included sport),” and “other.” We accordingly classified the responses into 3 levels of activity. A person replying “Yes” to the first question and “ADL” to the second was classified as having a limitation of ADL. A person replying “Yes” to the first question but not “ADL” to the second was classified as having a limitation of some activities except ADL. Respondents with other replies were classified as having no limitation of activities. Respondents admitted to hospitals, clinics, facilities for the elderly needing nursing care, and welfare facilities for the elderly requiring nursing care were considered to have a limitation of ADL.

### Calculation of expected years with and without activity limitation

Using the abovementioned data, we calculated the sex- and age-specific prevalences of ADL limitation and limitation of some activities except ADL in 1995, 1998, 2001, and 2004. The age groups were 0 to 4, 5 to 9, …, 80 to 84, and 85 years or older. The prevalence of persons admitted to hospitals and clinics in these years was estimated from those in 1996, 1999, 2002 and 2005 using linear interpolation and extrapolation.

Using the Sullivan method,^17^ we divided the expected years of life in age group *x* (*e_x_*) into those with and without activity limitation, as follows:

$$e_{x}=\sum \pi_{y}L_{y}/l_{x}+\sum \left(1-\pi_{y}\right)L_{y}/l_{x}$$

where ∑ represents the sum from age group *x* to the oldest age group in the age group of *y*, *π_y_* is the age-specific prevalence of activity limitation, *L_y_* is stationary population, and *l_x_* is the number of survivors in the life table. Also, we divided the expected years of activity limitation into those due to ADL limitation and those due to a limitation of some activities except ADL.

## RESULTS

Table 1 shows the prevalence of activity limitation by sex and age group in 1995 and 2004. In those aged 0 to 64 years, the prevalence of no activity limitation per 1000 population ranged from 922 to 936 among males and females in 1995 and 2004. In those 65 years or older, the prevalence of no activity limitation decreased, and the prevalence of limitation of some activities except ADL increased, from 1995 to 2004 in males and females. The prevalence of ADL limitation increased in females, but not in males.

**Table 1.** Prevalences of activity limitation in Japanese, by sex and age group (1995 and 2004)

	Age			
	
	<65 years		≥65 years	
		
	1995	2004	1995	2004
Males				
No activity limitation	936.4	931.7	730.3	711.5
Limitation of some activities except ADL	39.7	46.5	125.5	144.1
ADL limitation	24.0	21.7	144.2	144.4
Prevalence of ADL limitation among people:				
at home	16.3	15.7	95.6	96.7
admitted to hospitals or clinics	7.6	5.9	39.1	35.4
admitted to facilities for health of the elderly ​ who need nursing care	0.0	0.1	3.1	5.5
admitted to welfare facilities for elderly ​ who need nursing care	0.1	0.0	6.5	6.8

Females				
No activity limitation	930.7	921.6	697.1	667.6
Limitation of some activities except ADL	45.2	54.4	136.6	143.9
ADL limitation	24.2	24.0	166.3	188.5
Prevalence of ADL limitation among people:				
at home	18.1	19.2	101.6	117.0
admitted to hospitals or clinics	6.0	4.7	42.9	38.2
admitted to facilities for health of the elderly ​ who need nursing care	0.0	0.0	6.4	13.5
admitted to welfare facilities for elderly ​ who need nursing care	0.1	0.0	15.4	19.7

Prevalence is per 1000 population.

ADL: activities of daily living.

Table 2 shows the expected years with and without activity limitation, at birth and at 65 years of age, in males and females in 1995–2004. The number of expected years without activity limitation, at birth, in males was 68.5 in 1995, and increased to 69.7 in 2004. From 1995 to 2004, the number of expected years with limitation of some activities except ADL, and the number of expected years with an ADL limitation, at birth, increased from 4.2 to 5.1 and from 3.7 to 3.9, respectively. The proportion of expected years without activity limitation to total life expectancy at birth decreased from 89.7% to 88.6%. The proportion of expected years with limitation of some activities except ADL increased, but the proportion of those with ADL limitation did not. The number of expected years without activity limitation, at age 65 years, in males was 16.5 in 1995, and increased to 18.2 in 2004. The proportion of those years to total life expectancy at age 65 years decreased from 70.7% to 68.2%.

**Table 2.** Expected years with and without activity limitation, at birth and age 65 years, in Japanese males and females (1995–2004)

			Life expectancy	Expected years		

				without activity limitation	with limitation of some activities except ADL	with ADL limitation
Males	At birth	1995	76.38 (100.0)	68.49 (89.7)	4.18 (5.5)	3.72 (4.9)
		1998	77.16 (100.0)	68.83 (89.2)	4.25 (5.5)	4.08 (5.3)
		2001	78.07 (100.0)	69.48 (89.0)	4.73 (6.1)	3.85 (4.9)
		2004	78.64 (100.0)	69.66 (88.6)	5.05 (6.4)	3.93 (5.0)

	At age 65 years	1995	16.48 (100.0)	11.64 (70.7)	2.13 (12.9)	2.70 (16.4)
		1998	17.13 (100.0)	11.86 (69.3)	2.25 (13.2)	3.01 (17.6)
		2001	17.78 (100.0)	12.44 (70.0)	2.39 (13.5)	2.95 (16.6)
		2004	18.21 (100.0)	12.42 (68.2)	2.69 (14.8)	3.10 (17.0)

Females	At birth	1995	82.85 (100.0)	72.12 (87.1)	5.39 (6.5)	5.34 (6.4)
		1998	84.01 (100.0)	72.51 (86.3)	5.30 (6.3)	6.20 (7.4)
		2001	84.93 (100.0)	72.75 (85.7)	6.03 (7.1)	6.15 (7.2)
		2004	85.59 (100.0)	72.97 (85.3)	6.27 (7.3)	6.34 (7.4)

	At age 65 years	1995	20.94 (100.0)	13.85 (66.2)	2.89 (13.8)	4.20 (20.0)
		1998	21.96 (100.0)	14.18 (64.5)	2.80 (12.7)	4.99 (22.7)
		2001	22.68 (100.0)	14.47 (63.8)	3.10 (13.7)	5.11 (22.5)
		2004	23.28 (100.0)	14.67 (63.0)	3.30 (14.2)	5.31 (22.8)

Number of expected years as a proportion of life expectancy is shown as a percentage in parentheses.

ADL: activities of daily living.

The number of expected years without activity limitation, at birth, in females was 72.1 in 1995, and increased to 73.0 in 2004. From 1995 to 2004, the number of expected years with limitation of some activities except ADL, or with an ADL limitation, at birth, increased from 5.4 to 6.3 and from 5.3 to 6.3, respectively. The proportion of expected years without activity limitation to total life expectancy at birth decreased from 87.1% to 85.3%. The proportion of expected years with limitation of some activities except ADL and those with ADL limitation to total life expectancy both increased. The number of expected years without activity limitation, at age 65 years, in females was 20.9 in 1995, and increased to 23.3 in 2004. This represented a decrease from 66.2% to 63.0% of total life expectancy at age 65 years.

**Figure 1.**
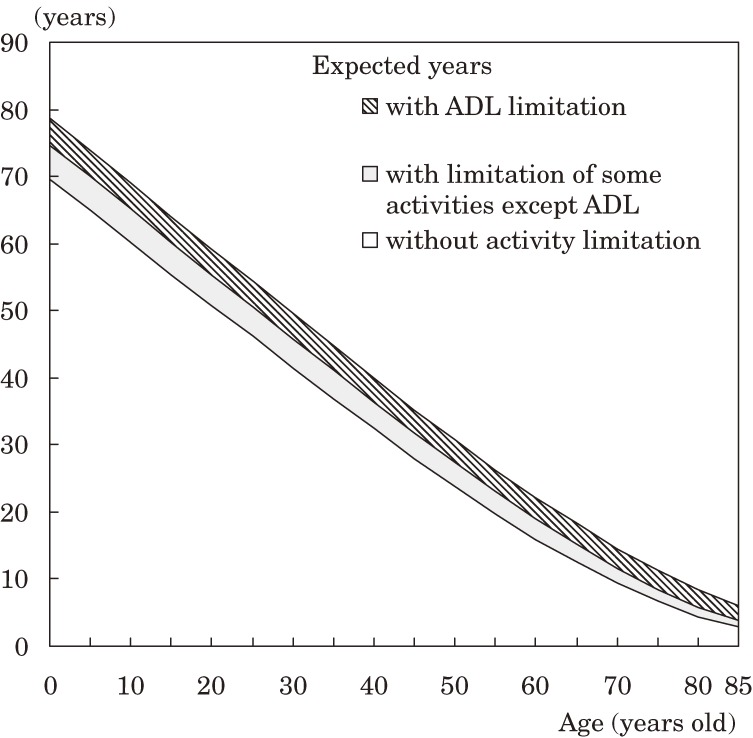
Number of expected years with and without activity limitation, by age, in Japanese males (2004).

**Figure 2.**
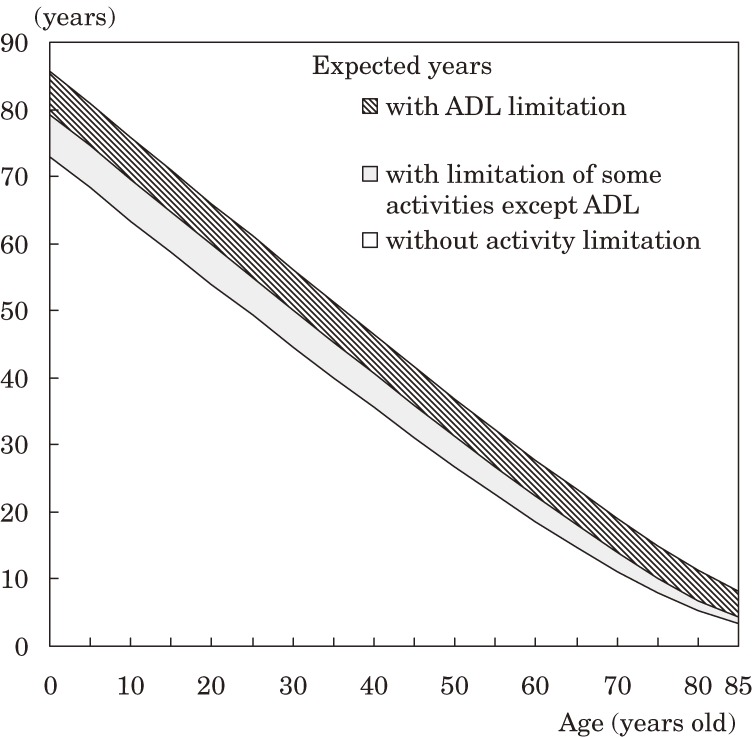
Number of expected years with and without activity limitation, by age, in Japanese females (2004).

Figures 1 and 2 show the number of expected years with and without activity limitation by age group in males and females, respectively, in 2004. The number of expected years without activity limitation rapidly decreased with age, but not in those with an ADL limitation.

## DISCUSSION

Life expectancy at birth in Japan is the longest in the world.^9^ In 2004, it was 78.6 years in males and 85.6 years in females.^11^ The aging population in Japan has increased substantially; the proportion of persons 65 years or older was 19.5% in 2004.^10^ Understanding the trends in disability-free life expectancy in such a population would prove useful for the discussion of means to assess population health.

This study presents a chronological series of the number of expected years of life with and without activity limitation in Japanese. From 1995 to 2004, the number of expected years without activity limitation, at birth, increased, and those years in persons with a limitation of some activities also increased. The proportion of the number of expected years without activity limitation to total life expectancy at birth decreased, and the proportion of expected years with a limitation of some activities except ADL increased. These results suggest that there was an increase in the duration of life with a light or moderate disability. An increase was reported after 1995 in the number of expected years lived in less-than-good self-rated health in Japan, as determined by observing trends in healthy life expectancy.^12^ Prolongation of the period of disability has been reported in some countries, while a decrease has been noted in others.^6^^–^^8^^,^^18^ We think that it is important to examine in more detail the factors related to these trends.^1^^,^^19^

In Japan, life expectancy is longer in females than in males. The proportion of expected years without activity limitation to total life expectancy was lower in females than in males in our population, as has been observed in many populations.^9^^,^^19^ During the period observed in our study, the life expectancy of females improved faster than that of males; however, the number of expected years of life without activity limitation increased more slowly among women than among males. The proportion of expected years with ADL limitation to total life expectancy at birth increased in females but not in males, which suggests that prolongation of severe disability might occur in females but not in males. Our study provides no information on the factors related to these sex differences.^20^

There were some limitations in the present study. We used data from representative national surveys in Japan.^10^^,^^11^^,^^14^^–^^16^ Although activity status in these surveys was measured in persons living at home by common instruments,^14^ it was self-reported and not comparable with measurements in previous studies.^6^^–^^8^ We used data for persons admitted to hospitals, clinics, facilities for the elderly needing nursing care, and welfare facilities for the elderly requiring nursing care; however, we did not include data for persons admitted to other facilities. We used the Sullivan method based on cross-sectional data to estimate the number of expected years with and without activity limitation. It would be useful to estimate these values using a multistate life table method based on longitudinal data.^21^ Unfortunately, these data are not available for a national representative population in many countries, including Japan. We did not estimate the standard errors of the expected years with and without activity limitation. To evaluate these parameters based on data from small samples, it would be necessary to consider their standard errors.^1^ The data used encompassed the 9-year period from 1995 to 2004. Observation over a longer period might be better for assessing trends in disability-free life expectancy.^1^ We observed the number of expected years with and without activity limitation in Japan. Comparison of our results with those from other populations should yield important information.^1^^,^^19^

In conclusion, the trends in the number of expected years with and without activity limitation suggest that, in Japanese males and females, the duration of life with light or moderate disability increased during the period from 1995–2004.
